# Salidroside promotes the repair of spinal cord injury by inhibiting astrocyte polarization, promoting neural stem cell proliferation and neuronal differentiation

**DOI:** 10.1038/s41420-024-01989-2

**Published:** 2024-05-09

**Authors:** Dingfei Qian, Yuan Dong, Xiaole Liu, Haichao Yu, Zelong Song, Chengqi Jia, Zhen Zhang, Shiqi Cao, Fanqi Hu, Xuesong Zhang

**Affiliations:** 1 Medical School of Chinese PLA, Chinese PLA General Hospital, 28 Fuxing Road, Haidian District, Beijing, 100853 China; 2 Department of Orthopedics, The Fourth Medical Center, Chinese PLA General Hospital, 51 Fucheng Road, Haidian District, Beijing, 100048 China; 3 CAS Key Laboratory for Biomedical Effects of Nanomaterials and Nanosafety & CAS Center for Excellence in Nanoscience, National Center for Nanoscience and Technology of China, Beijing, 100190 China; 4 Department of Orthopedics, Beijing Jishuitan Hospital, Capital Medical University, Beijing, 100035 China

**Keywords:** Spinal cord diseases, Neural stem cells

## Abstract

Spinal cord injury (SCI) remains a formidable challenge, lacking effective treatments. Following SCI, neural stem cells (NSCs) migrate to SCI sites, offering a potential avenue for nerve regeneration, but the effectiveness of this intrinsic repair mechanism remains suboptimal. Salidroside has demonstrated pro-repair attributes in various pathological conditions, including arthritis and cerebral ischemia, and the ability to curtail early-stage inflammation following SCI. However, the specific role of salidroside in the late-stage repair processes of SCI remains less defined. In this investigation, we observed that continuous salidroside treatment in SCI mice improved motor function recovery. Immunofluorescence-staining corroborated salidroside’s capacity to stimulate nerve regeneration and remyelination, suppress glial scar hyperplasia, reduce the activation of neurotoxic A1 astrocytes, and facilitate NSCs migration towards the injured region. Mechanistically, in vitro experiments elucidated salidroside’s significant role in restraining astrocyte proliferation and A1 polarization. It was further established that A1 astrocytes hinder NSCs proliferation while inducing their differentiation into astrocytes. Salidroside effectively ameliorated this inhibition of NSCs proliferation through diminishing c-Jun N-terminal kinase (JNK) pathway phosphorylation and restored their differentiation into neurons by suppressing the signal transducer and activator of transcription 3 (STAT3) pathway. In summary, our findings suggest that salidroside holds promise as a therapeutic agent for traumatic SCI treatment.

## Introduction

Spinal cord injury (SCI) stands as a formidable traumatic condition, marked by the potential for severe limb dysfunction, paraplegia, or complete paralysis. Regrettably, the realm of SCI therapeutics is still characterized by the absence of effective remedies [1, 2]. The intricate pathophysiological processes following SCI, collectively referred to as secondary injury, encompass inflammatory infiltration, oxidative stress, and glial scar formation, contributing to further functional impairment [3–5]. While hormonal interventions and certain small molecule drugs have been investigated to mitigate early-stage inflammatory responses [6, 7], their influence on the subsequent phases of SCI recovery has largely escaped scrutiny.

In recent years, research attention has increasingly gravitated toward glial scar formation in the late stages of SCI. Astrocytes, the predominant cell type in the central nervous system, become activated following injury, encasing the damaged region to limit the expansion of inflammation [8, 9]. Nonetheless, these glial scars persist, secreting chondroitin sulfate proteoglycans (CSPGs) and other factors, thereby erecting physical-chemical barriers that hinder nerve tissue repair in the late stages of SCI [10, 11]. Recent investigations have unveiled the existence of various astrocyte subtypes, including neurotoxic A1 astrocytes that manifest in the late stages of SCI and possess the capacity to promote inflammation and harm nerve cells [12, 13]. Our previous studies have indicated that neurotoxic A1 astrocytes induce neuronal apoptosis and inhibit axon outgrowth [14].

Within neural tissue, an intrinsic repair mechanism unfolds, where neural stem cells (NSCs) resident in the ependyma become activated following SCI and migrate to the injury site [15]. In the late stages of SCI, inflammatory infiltration and oxidative stress subside, theoretically allowing NSCs to survive, proliferate, and differentiate into neurons at the site of injury [16]. However, practical observations reveal that most NSCs differentiate into astrocytes, exacerbating the glial scar formation [15, 17]. Prior researches have confirmed that astrocytes contribute to NSC differentiation [18, 19]. Therefore, it is hypothesized that the emergence of neurotoxic A1 astrocytes disrupts the normal proliferation and neuronal differentiation of NSCs.

Salidroside, recognized as a small molecule prolyl-endopeptidase inhibitor, has demonstrated notable potential in restraining apoptosis and mitigating tissue inflammation, as indicated by previous studies [20–22]. In our prior research, salidroside emerged as a promising candidate for attenuating neuronal apoptosis triggered by spinal cord ischemia-reperfusion [23]. Additionally, salidroside administration has been associated with a reduction in functional deficits following SCI by curbing early inflammatory infiltration [24]. Salidroside can inhibit the activation of microglia/macrophages and the secretion of inflammatory factors through inhibiting NF-KB, MAPK, and ROS signaling pathways [25, 26]. Nevertheless, the effects of salidroside on astrocyte polarization and the fate of NSCs in the late stages of SCI remain insufficiently explored.

In our present study, we demonstrated that salidroside could enhance functional recovery in SCI mice by promoting axonal regeneration, myelin reconstitution, and inhibiting glial scar formation and the activation of neurotoxic A1 astrocytes. We also established that salidroside promoted the migration of NSCs to the injury site in vivo. In vitro experiments confirmed salidroside’s capacity to inhibit astrocyte proliferation and A1 polarization. Additionally, our results illuminated the role of neurotoxic astrocytes in suppressing NSC proliferation and inducing their differentiation into astrocytes, facilitated by the activation of c-Jun N-terminal kinase (JNK) and signal transducer and activator of transcription 3 (STAT3) pathways respectively, which could be restrained by salidroside through upregulating Sirt1. This study underscores the potential of salidroside as a promising therapeutic agent to enhance neural regeneration in SCI.

## Methods and materials

### Reagents and antibodies

The salidroside was purchased from MCE (HY-N0109, Monmouth Junction, NJ, USA). Primary antibodies used in this study included rabbit anti-STAT3 (ab68153, Abcam, Cambridge, UK), rabbit anti-p-STAT3 (ab76315, Abcam), mouse anti-Nestin (ab6142, Abcam), rabbit anti-Sox2 (ab92494, Abcam), rabbit anti-GFAP (ab7260, Abcam), mouse anti-MAP2 (ab11268, Abcam), rabbit anti-NeuN (ab177487, Abcam), rat anti-C3 (ab11862, Abcam), rabbit anti-GAP43 (ab75810, Abcam), mouse anti-Neurofilament (ab134306, Abcam), rabbit anti-β-actin (93473, Cell Signaling Technology, Danvers, MA, USA), mouse anti-JNK (66210-1-Ig, Proteintech, Chicago, USA), rabbit anti-p-JNK (ab4821, Abcam), rabbit anti-BrdU (ab152095, Abcam). Alexa Fluor 594 or 488 conjugated goat anti-mouse IgG (H + L) (115-545-062; 115-585-062) or goat anti-rabbit IgG (H + L) (111-545-045; 111-585-045), and Alexa Fluor 647 conjugated goat anti-rat IgG (H + L) (112-605-062) for immunofluorescence staining were purchased from Jackson ImmunoResearch (West Grove, PA, USA). Horseradish peroxidase (HRP)-conjugated goat anti-rabbit IgG (H + L) and HRP-conjugated goat anti-mouse IgG (H + L) for Western blot were purchased from Invitrogen (31460; 31430, Carlsbad, CA, USA). ELISA kits for IL-1β (88-5019-88) and IL-6 (88-7064-88) were purchased from Invitrogen. The CCK8 kits for cell viability were purchased from DOJINDO (CK04-1000t, Dojindo, Kumamoto, Japan). JSI-124 (HY-N1405) and SP600125 (HY-12041) were purchased from MCE.

### Contusive SCI mouse model

The conduction of all animal procedures strictly adhered to the Guidelines for the Care and Use of Laboratory Animals (National Institutes of Health) and approved by the Institutional Animal Care and Use Committee of National Center for Nanoscience and Technology (NCNST21-2101-YC01). Briefly, 45 female C57BL/6J mice (18–35 g; 8 weeks old) underwent deep anesthesia via intraperitoneal injection of pentobarbital sodium (50 mg/kg of body weight). Following this, the dorsal fur of the mice was meticulously removed and disinfected with an iodophor solution. Subsequently, the T10 lamina was excised, revealing the spinal cord. After the dorsal surface of the spinal cord was fully exposed, a rod (1.3 mm in diameter; RWD Life Science Corp., C4p01-001, China) was used to compress the spinal cord with a force of 50 kdynes and a dwell time of 10 s. After surgery, the muscular and dermal layers were meticulously sutured. The mice received daily abdominal massages to facilitate defecation and urination until the restoration of reflexive micturition control. These SCI models were randomly segregated into three groups: a SCI-only group (*n* = 25/group), a low-dose salidroside treatment group (*n* = 25/group) and a high-dose salidroside treatment group (*n* = 25/group). The number of standardized animal models could meet statistical needs as reported [7]. No mice died before the end of the experiment, and all model mice were included in the final analysis. The SCI-only group functioned as the control. The SCI-only group functioned as the control. Salidroside was administered via intraperitoneal injection at a dosage of 50 mg/kg (low-dose) or 100 mg/kg (high-dose) once every 2 days until the point of sacrifice. For late-stage drug therapy experiments (*n* = 30/group), intraperitoneal injection of salidroside (100 mg/kg) commenced on the 16th day after surgery, along with colivelin (Col) (STAT3 agonist, 1 mg/kg, i.p.) or anisomycin (Ani) (JNK agonist, 15 mg/kg, s.c.), every two days until euthanasia.

### Assessment of locomotor capacity

The evaluation of mouse locomotor capacity post-SCI was conducted through Basso Mouse Score (BMS) assessments on days 1, 3, 7,14, 21, and 28 after surgery. Scores range from 0 for complete paraplegia to 9 for normal function.

### Footprint analysis

Gait and motor coordination were assessed at 28th day post surgery. The forelimbs were marked with blue dye, while the hindlimbs were marked with red dye. Mice were placed on a track lined with blotting paper, with an incentive cassette placed at the end to encourage rapid traversal. Footprint patterns were digitized, and representative images were employed to assess coordination.

### Rotarod test

Mice were positioned on a rotating rod that experienced gradual acceleration. The duration and rotational speed when the mice fell down were recorded. Prior to the official experiment, adaptation trials were conducted for two days, with each mouse undergoing two tests.

### Drug safety evaluation

In an independent experiment, SCI models were randomly allocated into four groups (*n* = 4/group), each receiving varying concentrations of salidroside. Subsequently, the mice were euthanized, and blood samples were collected for serological analysis. Additionally, major organs were dissected for hematoxylin-eosin (H&E) staining to assess histological changes.

### Tissue processing

On days 7, 14, 21, and 28 post injury, the mice were humanely euthanized through intraperitoneal administration of pentobarbital sodium (80 mg/kg). The hearts of the mice underwent immediate perfusion with 20 mL of ice-cold saline followed by 10 mL ice-cold paraformaldehyde (4%, w/v). The incision of SCI was opened, and the spinal cord segment encompassing the lesion site was extracted. Spinal cord samples were subsequently fixed in paraformaldehyde (4%, w/v) at 4 °C for 24 h, before dehydrated in a sucrose-PBS gradient (20% and 30%, w/v). Finally, the samples were embedded in OCT and sectioned into continuous longitudinal sections of 10 µm. All sections were stored at −80 °C for subsequent immunostaining.

### Primary astrocyte culture and treatment

Primary astrocytes were obtained from the brains of 1~3-day-old C57BL/6J mice according to an established protocol [27]. Astrocytes were cultured in T75 flasks pre-coated with poly-L-lysine (Sigma, St.Louis, MO, USA) to obtain primary mixed glial cell cultures using DMEM/F12 (Gibco, Carlsbad, CA, USA) supplemented with 10% heat-inactivated fetal bovine serum (FBS, Gibco), 1% penicillin and streptomycin solution (Gibco), 2 mM l-glutamine (Gibco), 100 µM non-essential amino acids (Gibco), and 2 mM sodium pyruvate (Gibco). Subsequent to confluence, the cultures underwent agitation at 180 rpm for 30 min on an orbital shaker to eliminate microglia. The mixed glial cultures were further treated with 20 mL of fresh culture medium and subjected to shaking at 240 rpm for 6 h, an action that effectively removed oligodendrocyte precursor cells (OPCs). The ensuing astrocytes were employed for subsequent experiments. To induce neurotoxic A1 astrocytes, a mixture termed “Activator” comprising IL-1α (3 ng/mL, I3901, Sigma), TNFα (30 ng/mL, 8902SF, Cell Signaling Technology), and C1q (400 ng/mL, MBS143105, MyBioSource, San Diego, Southern California, USA) was concurrently added to the medium, with cultures maintained for 24 h [12]. Following this, the supernatant of neurotoxic A1 astrocytes was replaced with fresh complete medium and continued for an additional 24 h. The resultant supernatant (astrocyte conditioned medium, ACM) was collected for subsequent NSC intervention experiments (Supplementary Fig. 1).

### Primary NSCs culture and treatment

Primary NSCs were isolated from the brains of 1~3-day-old C57BL/6J mice using a process resembling the procedure for astrocytes, except that the cell precipitates were resuspended in NSCs growth medium [28]. This growth medium consisted of DMEM/F12 (Gibco) supplemented with 2% N2 (Gibco), 1% B27 (R&D Systems, Minneapolis, MN, USA), bFGF 20 ng/ml (R&D Systems), and EGF 20 ng/ml (R&D Systems). NSCs were identified by Nestin (red) and Sox2 (green) (Supplemental Fig. 2). Differentiation experiments necessitated the substitution of the medium with neuron complete medium, characterized by Neurobasal medium (Gibco) supplemented with 2% B27 (Gibco), 1% l-glutamine (Gibco), 100 IU/ml penicillin, and 100 mg/ml streptomycin. For dispersed cells, neurospheres were digested with Accutase (Gibco) and seeded within well plates pre-coated with poly-L-lysine (Sigma). In cell proliferation experiments, neurospheres or dispersed NSCs were cultivated in NSCs growth medium supplemented with ACM in the presence or absence of Sal (100 µM), JSI-124 (0.5 µM), or SP600125 (30 µM). To assess differentiation, dispersed NSCs were cultivated in neuron complete medium featuring the aforementioned stimulating agents.

### Cell viability

The viability of primary astrocytes and NSCs was evaluated with a CCK8 assay (Dojindo). After 24 h of incubation with varying concentrations of salidroside, with or without Activator or ACM, wells were rinsed 3 times with PBS, followed by the addition of CCK8 solution (10 μL; 1:10 dilution) in fresh culture medium (100 μL) and then incubated for 1 h at 37 °C. The optical absorbance was measured at 450 nm using a microplate reader (ELx800; Bio-Tek, Winooski, VT, USA).

### Western blot analysis

Proteins were extracted from cells using a protein extraction kit (Solarbio, Beijing, China). Protein concentration was quantified via a BCA assay kit (Thermo Scientific, Waltham, MA, USA). 20 μg of protein samples underwent separation by SDS-PAGE, transferring to PVDF membranes (ISEQ00010, Millipore), and then blocking with 5% bovine serum albumin (BSA, Solarbio). Following this, the membranes were incubated with primary antibodies overnight at 4 °C. Thereafter, HRP-conjugated secondary antibodies were introduced at room temperature for 2 h. Reacting bands were visualized using ECL reagent (170-5061, Bio-Rad, Hercules, CA, USA), with protein band densities semi-quantified via Image J (National Institutes of Health, Bethesda, MD, USA).

### Immunofluorescence staining

Spinal cord sections or cultured cells were fixed with pre-cooled 4% paraformaldehyde for 15 min at 4 °C, then permeabilized with 0.3% Triton X-100 for 20 min, and blocked with 5% BSA for 1 h. The samples were incubated at 4 °C overnight with primary antibodies, followed by incubation with the corresponding fluorescent secondary antibody for 2 h at room temperature. After triple washes with PBS, the nuclei were stained with DAPI (Thermo Fisher Scientific), and fluorescent images were captured using an epifluorescence microscope (AxioVertA1 and ImagerA2), or a confocal fluorescence microscope (LSM710; Carl Zeiss, Germany).

### Nissl staining of spinal cord slices

Nissl staining in spinal cord samples was performed on the 14th day post surgery. Subsequent to rinsing the slices with distilled water, they were immersed in a cresol violet solution for 10 min. Then slices were rinsed with distilled water, differentiated with 95% ethanol, rinsed with xylene, and finally fixed with neutral balsam. Bright-field images were acquired using an epifluorescence microscope (AxioVertA1 and ImagerA2).

### Real-time reverse transcription polymerase chain reaction (RT-qPCR)

Total RNA was extracted from astrocytes or NSCs post-various treatments using Trizol Reagent (Invitrogen) according to the manufacturer’s instructions. The concentrations of RNA were gauged by a Biometra Optical Thermocycler (Analytik Jena, Goettingen, Germany). Five hundred nanograms of RNA underwent reverse transcription into cDNA employing the High-capacity cDNA Reverse Transcription Kit (Thermo Fisher Scientific). The primer sequences used for qPCR amplification were:

C3: 5′-ACATGC GCCGAA GCA GA-3′ (forward)

 5′-ACCCGGACCTCAAAA CTGG-3′ (reverse)；

GAPDH: 5′-TGTGATGGGTGTGAACCACG-3′ (forward)

 5′-CAGTGAGCTTCCCGTTCACC-3′ (reverse)；

NeuN: 5′-GACAACCAGCAACTCCACCC-3′ (forward)

 5′-GAGCCCCGCTCGTTAAAAAT-3′ (reverse)；

GFAP: 5′-CATGCCACGCTTCTCCTTGT-3′ (forward)

 5′-ATCATCTCTGCACGCTCGCT-3′ (reverse)；

iNOS: 5′-ACGGACGAGACGGATAG-3′ (forward)

 5′-GGGCTTCAAGATAGGGA-3′ (reverse)；

Nestin: 5′AAGCAGGGTCTACAGAGTCAGATCG-3’ (forward)

 5′-GCTGTCACAGGAGTCTCAAGGGTAT-3’ (reverse)；

Quantitative real-time PCR was performed using SYBR qRCR premix (Takara, Kyoto, Japan). Cycling conditions encompassed an initial denaturation at 95 °C for 30 s, followed by 40 cycles at 95 °C for 5 s, 60 °C for 30 s, and 72 °C for 10 min. Target gene expression was normalized to GAPDH expression using the ∆∆CT method.

### Enzyme-linked immunosorbent assay

The concentrations of IL-1β and IL-6 in ACM post-various treatments were quantified using ELISA kits according to the manufacturers’ protocols. Briefly, the ACM was collected and centrifuged at 2000 rpm for 20 min to eliminate debris. 100 µL of all samples or standards were added to appropriate wells of capture-antibody pre-coated 96 well plates for 1 h incubation followed by 100 µL of detection antibody for another 1 h. Then all wells underwent three washes with 1× wash buffer followed by 100 µL/well of TMB substrate for 10 min in darkness. Finally, STOP Solution (0.16 M H_2_SO_4_) was added and absorbance was read at 450 nm wavelengths using a microplate reader (ELx800, Bio-Tek, USA) within 30 min.

### Statistical analyses

Results are presented as mean ± SD of at least three independent experiments. Means were compared by one-way ANOVA followed by Bonferroni’s post hoc tests for multiple comparisons (SPSS 21; SPSS, Chicago, IL, USA). *P* values of <0.05 (two tailed) were considered statistically significant. *p < 0.05; **p < 0.01; ***p < 0.001.

## Results

### Long-term salidroside treatment promoted functional recovery after SCI

**Fig. 1:**
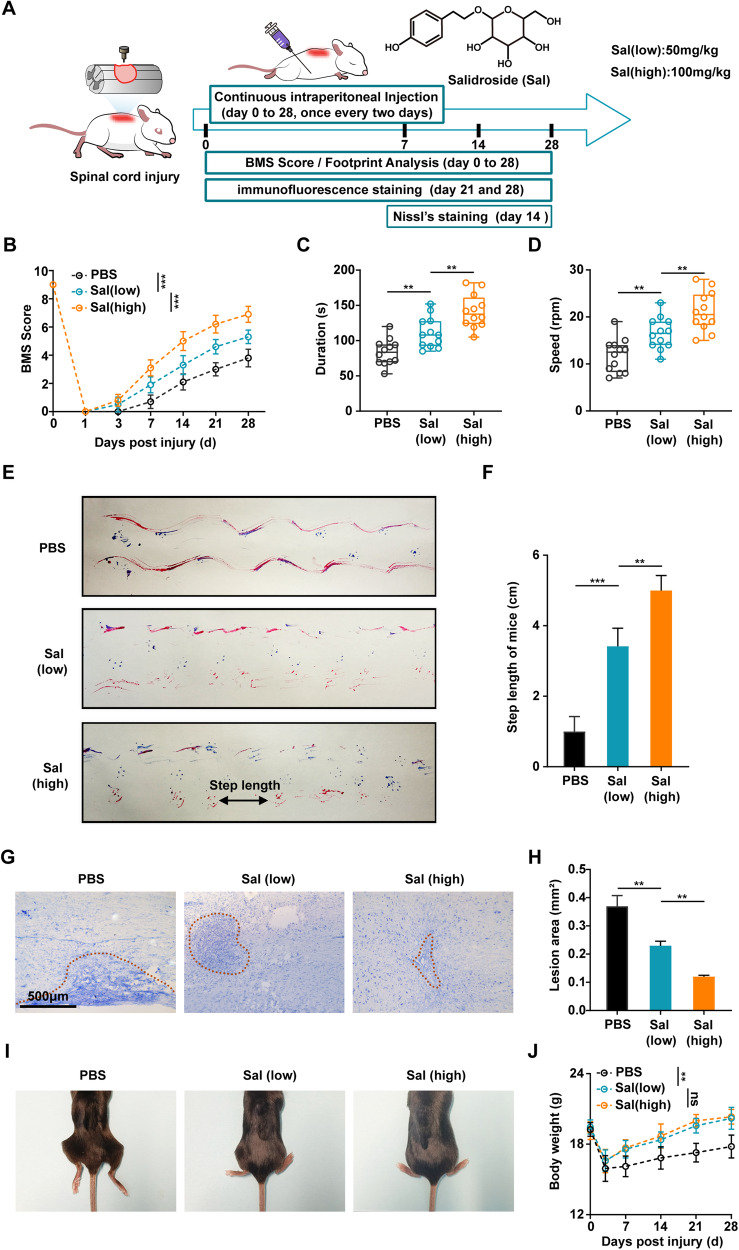
Salidroside treatment promoted functional recovery after SCI. **A** Experimental design schematic: Salidroside (Sal) was intraperitoneally administered within 15 min after surgery and subsequently injected every two days until mice were sacrificed. **B** Basso mouse score (BMS) assessed motor function recovery at different time points after SCI. **C** Rotarod tests performed on day 28 post injury to measure the duration of mice maintaining balance on the rotarod. **D** Rotational speed at which mice fell in the rotarod test **E** Representative footprints of mice 28 days after SCI, with blue denoting forepaw prints and red indicating hindpaw prints. **F** Quantitative footprint analysis. **G** Representative Nissl-stained results of spinal cord on day 14 post injury, delineating the lesion boundary with dashed lines. Scale bar, 500 μm. **H** Quantitative lesion area analysis. **I** Photographs of mice’s lower bodies 28 days post injury. **J** Body weight of mice with SCI. All data are presented as means ± SD (*n* = 5 mice per group). *p < 0.05; **p < 0.01; ***p < 0.001.

To assess the efficacy of salidroside in vivo, motor function evaluations and histological examinations were performed in mice subjected to weight-induced spinal contusion at the T10 level (Fig. 1A). Long-term salidroside treatment resulted in a significant improvement in the motor function scores of SCI-afflicted mice, with higher salidroside dose yielding more pronounced benefits (Fig. 1B). Within the treatment group, mice exhibited enhanced lower limb strength and improved balance (Fig. 1C, D). Notably, footprint analysis revealed that the treated mice exhibited partial restoration of lower limb walking function, with an increased stride distance (Fig. 1E, F). Salidroside treatment also demonstrated its effectiveness in reducing the size of the SCI-affected region, as evident from Nissl staining of the spinal cord (Fig. 1G, H). Importantly, the observed therapeutic effects were augmented with a higher salidroside dose. Furthermore, the treated mice displayed improved overall metabolic status, characterized by the absence of lower limb muscle atrophy and stiffness and a more rapid recovery of body weight (Fig. 1I, J). In summary, these findings collectively suggest that salidroside promotes the recovery of motor function in the context of SCI.

### Long-term salidroside treatment promoted tissue recovery after SCI

**Fig. 2:**
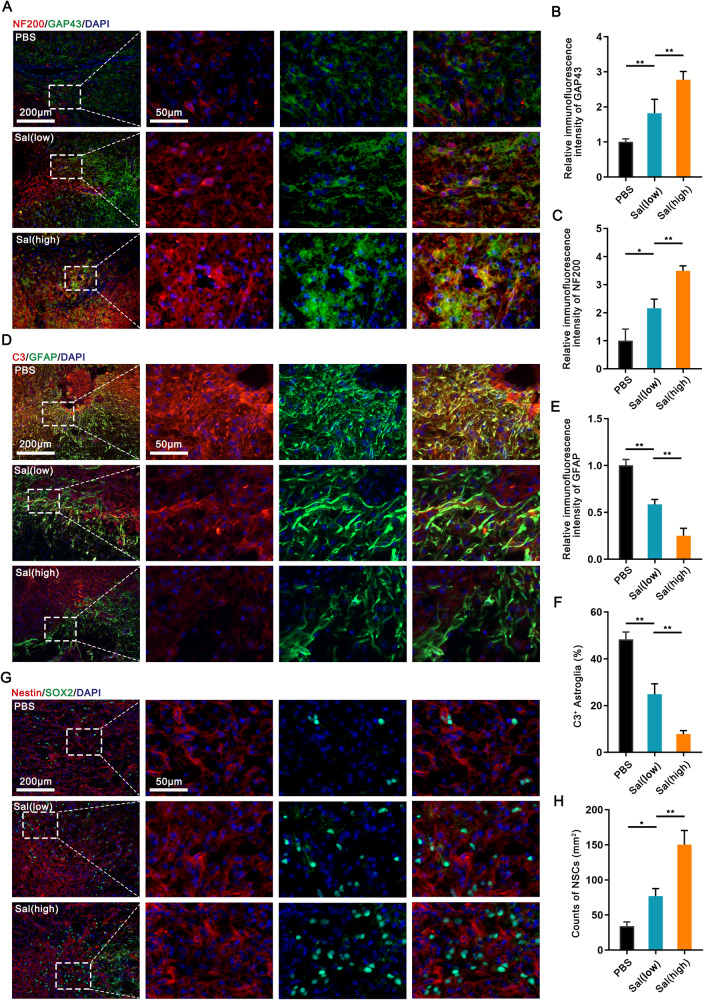
Salidroside promoted tissue repair after SCI. **A** Immunofluorescence images of NF200 (red) and GAP43 (green) on day 28 after injury in spinal cord lesion areas. All nuclei were stained with DAPI (blue). Scale bar, 200 μm. **B**, **C** Semi-quantification of NF200 and GAP43 intensity in (**A**). **D** Glial scar images detected by GFAP (green) and C3 (red) immunohistochemical staining on the 21st day post injury in spinal cord lesion areas. All nuclei were counterstained with DAPI (blue). Scale bar, 200 μm. **E** Semi-quantification of GFAP intensity in (**D**). **F** Quantitative analysis of the ratio of C3 + / GFAP+ cells around the lesion areas in (**D**). **G** Images of NSCs migrating into lesion areas on the 21st day post injury in spinal cord lesion areas. NSCs were double-positive for Nestin (red) and Sox2 (green). All nuclei were counterstained with DAPI (blue). **H** Quantification of NSCs in (**G**). All data are presented as means ± SD (*n* = 5 mice per group). *p < 0.05; **p < 0.01; ***p < 0.001.

To investigate whether salidroside treatment could stimulate nerve regeneration, spinal cord sections from mice sacrificed on the 28^th^ day after surgery were subjected to histological analysis, specifically involving the assessment of growth-associated protein-43 (GAP43) and neurofilament (NF200). As illustrated in Fig. 2A, salidroside treatment elicited a significant upregulation in the expression of GAP43, signifying enhanced nerve regeneration and the emergence of newborn neurons (Fig. 2B). Furthermore, NF200, a widely used indicator of axon elongation, exhibited a notable elevation (Fig. 2C). Concurrently, salidroside treatment fostered remyelination, evidenced by heightened myelin basic protein (MBP) expression at the margins of lesioned areas, with preserved myelinated axons in the adjacent regions surrounding the injury site (Supplemental Fig. 3).

In addition to the remarkable progress in nerve repair, our investigation focused on the impact of salidroside treatment on glial scar formation and NSCs within the injury site. On the 21st day post injury, spinal cord sections were stained, revealing that salidroside treatment led to a significant reduction in the density of glial scar formation, a characteristic assessed through glial fibrillary acidic protein (GFAP) staining, and a decrease in the levels of neurotoxic A1 astrocyte polarization, as indicated by Complement C3 (C3) staining, within the injured region (Fig. 2D–F). Moreover, a substantial increase in the number of NSCs, as denoted by double-positive staining for Nestin and Sox2, was detected within the injury site (Fig. 2G, H). This increase in NSCs is likely to contribute to the improved recovery of nerve regeneration, as presented in Fig. 2A. Intriguingly, salidroside demonstrated inhibition of M1 polarization post-SCI, as reported. However, no discernible differences in the rate of iNOS-positive microglia/macrophages (Supplemental Fig. 4A, B) and iNOS mRNA levels (Supplemental Fig. 4C) were noted between different dose groups. This suggests that high-dose salidroside may exert a more pronounced promoting effect on the later stages of regeneration and repair processes. It is imperative to note that further escalation of the therapeutic dose may pose a risk of toxicity. Immunohistochemical and serological tests revealed that an excessive dose (200 mg/kg) induced damage to the liver and spleen of mice (Supplemental Fig. 5). Clearly, our long-term administration of salidroside significantly contributed to tissue recovery within the injured region of SCI in mice.

### Salidroside inhibited the proliferation and polarization of astrocytes ex vivo

**Fig. 3:**
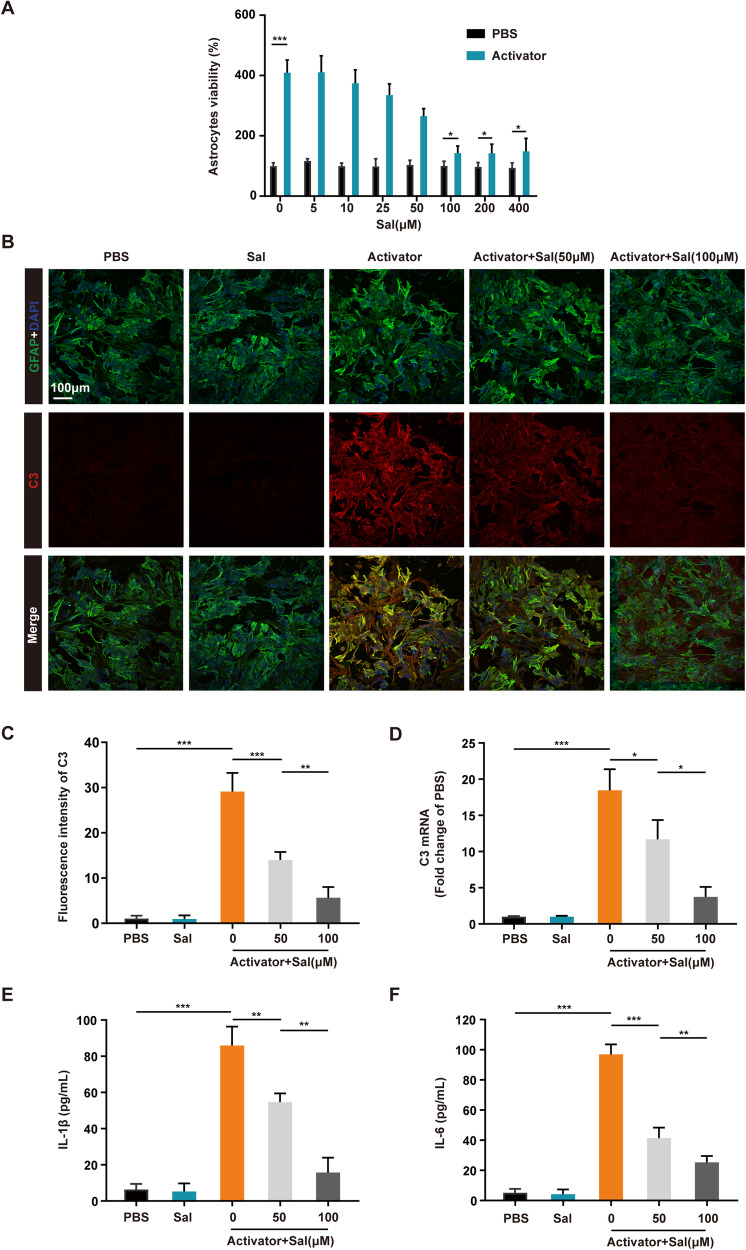
Salidroside inhibited the proliferation and polarization of astrocytes ex vivo. **A** Cell viability assay of primary astrocytes after various treatments for 24 h. **B** Immunofluorescence images of C3 (red) and GFAP (green) after different treatments to detect astrocyte polarization. All cell nuclei were stained with DAPI (blue). Scale bar, 100 μm. **C** Semi-quantification of C3 intensity in (**B**). **D** qRT-PCR of C3 mRNA expression in astrocytes from (**B**). (ELISA results of IL-1β (**E**) and IL-6 (**F**) in astrocyte supernatant. All data are presented as means ± SD (*n* = 6 / group). *p < 0.05; **p < 0.01; ***p < 0.001.

Previous researches have demonstrated that reactivated astrocytes can undergo further differentiation into neurotoxic A1 astrocytes when subjected to various factors, most notably TNF-α, IL-1α, and C1q [12, 13]. A1 astrocytes lose their normal supportive function to neurons and instead contribute to ongoing neural tissue damage [29–31]. Our previous investigation established that small molecule drugs, such as the Notch pathway inhibitor DAPT, can inhibit the A1 polarization of astrocytes [14]. In an effort to confirm these findings observed in vivo, we isolated primary astrocytes and subjected them to a mixture of TNF-α, IL-1α, and C1q, referred to as the “Activator”, to induce astrocyte proliferation and polarization, as documented in earlier studies. Remarkably, our observations indicated that salidroside did not exert significant cytotoxicity on astrocytes and instead hindered the proliferation of astrocytes induced by the Activator, with the most pronounced inhibitory effect occurring at a concentration around 100 μM (Fig. 3A). Immunofluorescence analysis revealed that salidroside reduced the expression of C3 protein, a distinctive marker of neurotoxic astrocytes (Fig. 3B, C). Furthermore, PCR results demonstrated a reduction in the gene transcription levels of C3 with salidroside treatment (Fig. 3D). Additionally, salidroside decreased the levels of inflammatory factors secreted by neurotoxic astrocytes, signifying a reduction in the toxicity of these astrocytes (Fig. 3E, F). In conclusion, salidroside effectively inhibits the proliferation and polarization of astrocytes, thereby diminishing their interference with nerve regeneration.

### Salidroside mitigated the proliferation inhibition of NSCs induced by neurotoxic astrocytes ex vivo

**Fig. 4:**
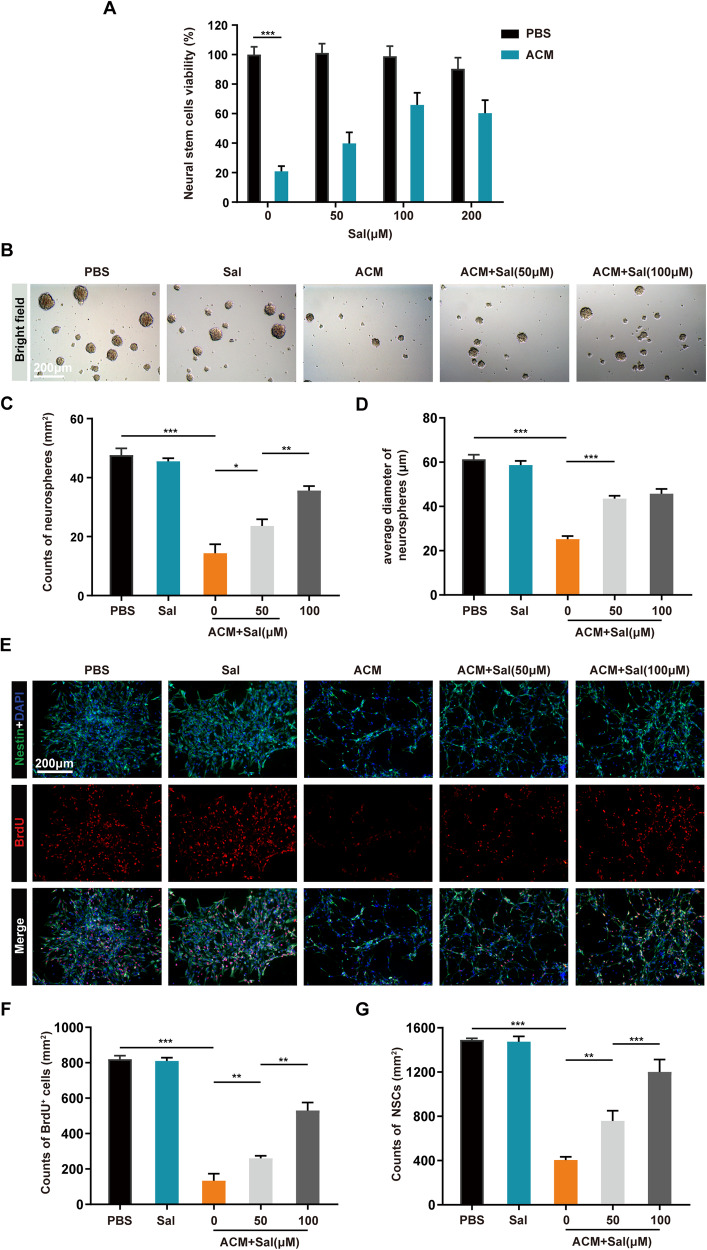
Salidroside mitigated the proliferation inhibition of NSCs induced by neurotoxic astrocytes ex vivo. **A** Cell viability assay of primary NSCs after different treatments for 24 h. **B** Bright-field images of neurospheres following various treatments. Scale bar, 200 μm. **C** Quantitative analysis of neurospheres counts in (**B**). **D** Quantitative analysis of the average neurospheres diameter in (**B**). **E** Immunofluorescence images of BrdU (red) and Nestin (green) following different treatments to assess NSC proliferation. All cell nuclei were stained with DAPI (blue). Scale bar, 200 μm. **F** Quantitative analysis of BrdU^+^ cell density in (**E**). **G** Quantitative analysis of total cell density in (**E**). All data are presented as means ± SD (*n* = 6 / group). *p < 0.05; **p < 0.01; ***p < 0.001.

NSCs exhibit greater resilience to external stimuli in comparison to mature neurons. When damaged, they are prone to slower proliferation, or even arrested proliferation, and premature differentiation, as opposed to the immediate apoptosis typically seen in neurons [32–34]. To delve deeper into the effects of salidroside on NSCs, we isolated primary NSCs and exposed them to the culture supernatant of neurotoxic astrocytes (astrocyte conditioned medium, ACM) for NSCs intervention. Results from cell viability assays demonstrated that ACM significantly impeded the proliferation of NSCs, and the application of salidroside partially alleviated this inhibition of proliferation (Fig. 4A). Similarly, bright-field observations of neurospheres indicated that ACM resulted in a reduction in the number and size of neurospheres, whereas salidroside restored normal neurosphere proliferation to some extent (Fig. 4B–D). Immunofluorescence results further revealed that salidroside mitigated the proliferation inhibition of NSCs induced by ACM, as evidenced by the presence of more BrdU-positive cells and increased NSC density (Fig. 4E–G). These results collectively highlight the ability of salidroside to diminish the proliferation inhibition of NSCs induced by neurotoxic astrocytes.

### Salidroside inhibited astrocytic differentiation of NSCs induced by neurotoxic astrocytes ex vivo

**Fig. 5:**
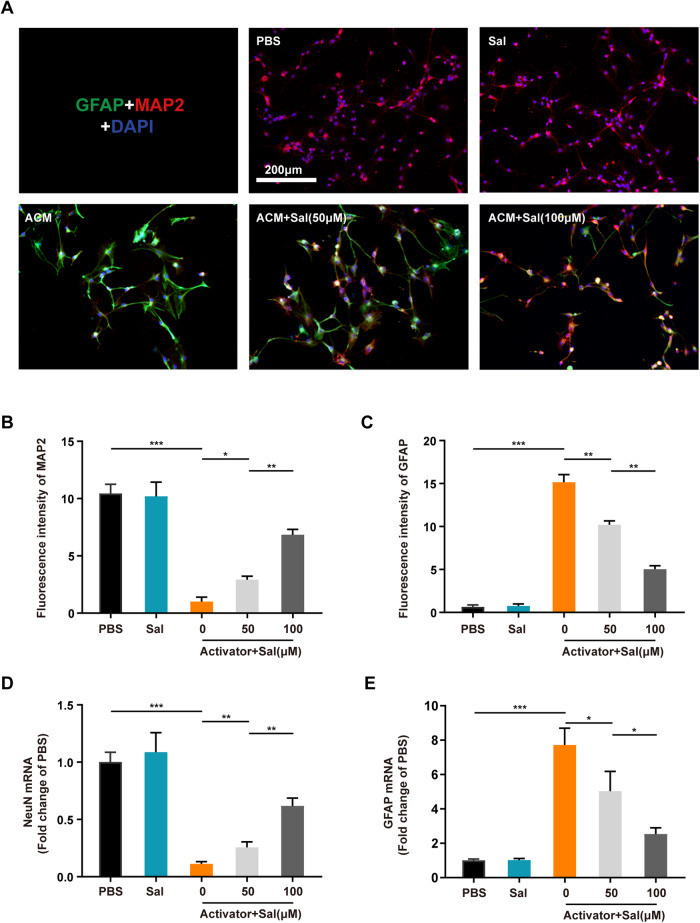
Salidroside inhibited astrocytic differentiation of NSCs induced by neurotoxic astrocytes ex vivo. **A** Images of MAP2 (red, marker of neurons) and GFAP (green, marker of astrocytes) immunofluorescence staining of NSCs after 24-h incubation with ACM ± different salidroside (Sal) concentrations. All cell nuclei were stained with DAPI (blue). Scale bar, 200 μm. **B** Semi-quantification of MAP2 intensity in (**A**). (**C**) Semi-quantification of GFAP intensity in (**A**). mRNA expression levels of NeuN (**D**) and GFAP (**E**) in NSCs from (**A**). All data are presented as means ± SD (*n* = 6 / group). *p < 0.05; **p < 0.01; ***p < 0.001.

Following SCI, NSCs theoretically have the capacity to migrate from the ependyma to the injured region and differentiate into neurons, thereby facilitating the reestablishment of axonal connections—a pivotal endogenous repair mechanism in nerve injuries [15, 16]. However, the practical outcomes of this endogenous repair process have been far from ideal. Studies have suggested that a significant portion of NSCs migrating to the injured area differentiate into astrocytes, thereby exacerbating the formation of the glial scar [17, 32]. Our investigation revealed that neurotoxic astrocytes can prompt the differentiation of NSCs into astrocytes, even in the presence of neuronal differentiation induction medium. This differentiation is evident through an increase in GFAP fluorescence intensity and a decrease in microtubule-associated protein 2 (MAP2) fluorescence intensity, which could be effectively mitigated by salidroside treatment (Fig. 5A–C). Additionally, PCR results indicated that salidroside decreased the heightened levels of GFAP transcripts in NSCs while restoring the transcript levels of neuronal marker NeuN (Fig. 5D, E). These findings collectively suggest that salidroside effectively inhibits the astrocyte-oriented differentiation of NSCs induced by neurotoxic astrocytes.

### Salidroside affected the proliferation and differentiation of NSCs through JNK and STAT3 pathways ex vivo

**Fig. 6:**
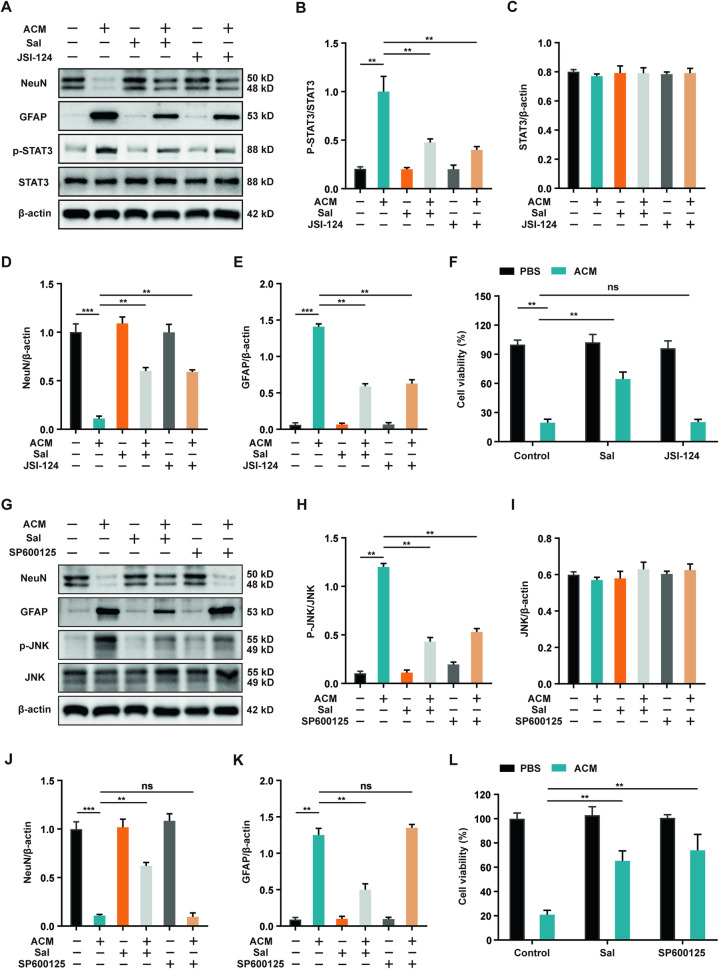
Salidroside affected the proliferation and differentiation of NSCs through JNK and STAT3 pathways ex vivo. **A** Western blot bands displaying total protein levels (STAT3, NeuN, and GFAP) and phosphorylated protein (p-STAT3) in NSCs under ACM stimulation for 24 h with or without Sal (100 μM) or JSI-124 (0.5 μM). **B**–**E** Semi-quantitative analysis of total protein levels (STAT3, NeuN, and GFAP) normalized to β-actin and phosphorylated STAT3 levels (p-STAT3/STAT3) in (**A**). **F** Cell viability assay of primary NSCs under ACM stimulation for 24 h in the presence or absence of Sal (100 μM) or JSI-124 (0.5 μM). **G** Western blot bands displaying total protein levels (JNK, NeuN, and GFAP) and phosphorylated protein (p-JNK) in NSCs under ACM stimulation for 24 h with or without Sal (100 μM) or SP600125 (30 μM). **H**–**K** Semi-quantitative analysis of total protein levels (JNK, NeuN, and GFAP) normalized to β-actin and phosphorylated JNK levels (p-JNK/JNK) in (**G**). **L** Cell viability assay of primary NSCs under ACM stimulation for 24 h in the presence or absence of Sal (100 μM) or SP600125 (30 μM). All data are presented as means ± SD (*n* = 6/group). *p < 0.05; **p < 0.01; ***p < 0.001.

The STAT3 pathway is intrinsically linked to the process of cell differentiation [35–37]. In this study, western blot results corroborated that ACM induced the activation of the STAT3 pathway in NSCs, subsequently enhancing the expression of GFAP and diminishing the expression of NeuN. Importantly, salidroside effectively inhibited the ACM-induced activation of the STAT3 pathway in NSCs. The STAT3 phosphorylation inhibitor JSI-124 also successfully restrained the astrocytic differentiation of NSCs in a similar manner (Fig. 6A–E). It is noteworthy, however, that the intervention of the STAT3 pathway had no discernible impact on cell proliferation, as indicated by cell viability assays (Fig. 6F). This suggests that the proliferation of NSCs is regulated by alternative pathways. Previous studies have elucidated the inhibitory effect of IL-1β on the proliferation of stem cells through the activation of the JNK pathway [32]. Moreover, our study identified the secretion of IL-1β by neurotoxic astrocytes. We therefore investigated the expression of the JNK pathway in NSCs, which did not significantly affect NSCs differentiation (Fig. 6G–K). However, it was evident that the elevation of JNK phosphorylation induced by ACM significantly inhibited the proliferation of NSCs. This inhibitory effect was alleviated by salidroside treatment or SP600125 (a JNK phosphorylation inhibitor) (Fig. 6L). Collectively, our results illustrate that salidroside modulates the proliferation and differentiation of NSCs through the JNK and STAT3 pathways, respectively.

### Salidroside affected the proliferation and differentiation of NSCs through JNK and STAT3 pathways in vivo

**Fig. 7:**
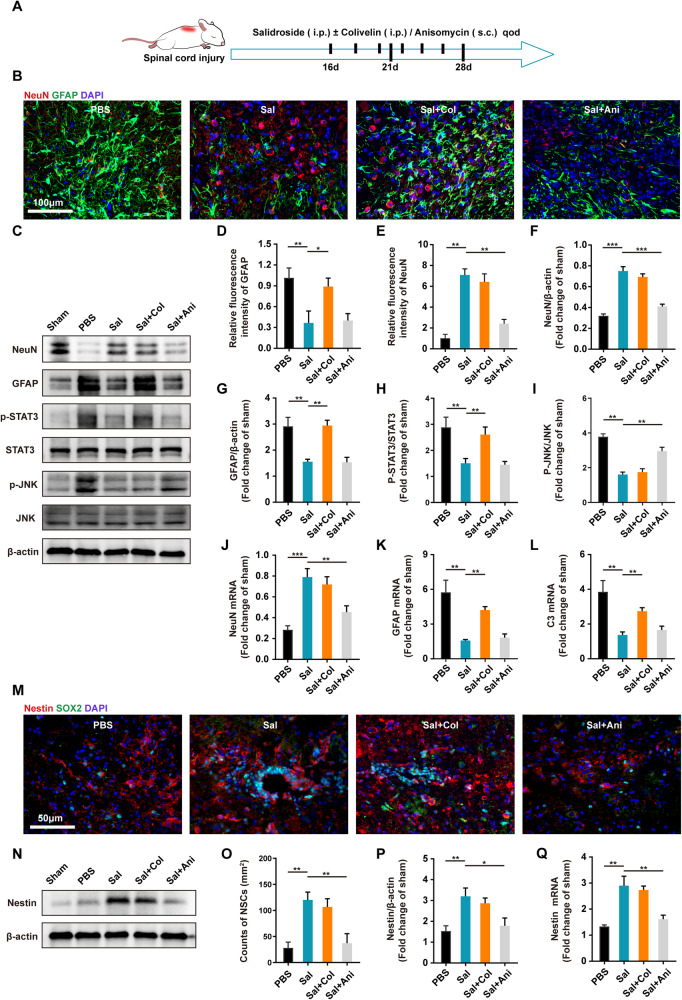
Salidroside affected the proliferation and differentiation of NSCs through JNK and STAT3 pathways in vivo. **A** Experimental design diagram: Salidroside (Sal) (100 mg/kg) was administered intraperitoneally from 16th day after surgery, along with Colivelin (Col) (1 mg/kg, i.p.) or Anisomycin (Ani) (15 mg/kg, s.c.), every 2 days until the mice were euthanized. **B** Immunofluorescence images of NeuN (red) and GFAP (green) on day 28 after injury in spinal cord lesion areas. All nuclei were stained with DAPI (blue). Scale bar, 100 μm. **C** Western blot bands displaying total protein levels (STAT3, JNK, NeuN, and GFAP) and phosphorylated protein (p-STAT3, p-JNK) of spinal cord from mice 28^th^ day after injury. **D**–**E** Semi-quantitative analysis of GFAP and NeuN intensity in (**B**). **F**–**I** Semi-quantitative analysis of total protein levels (NeuN and GFAP) normalized to β-actin and phosphorylated STAT3 and JNK levels in (**C**). mRNA expression levels of NeuN (**J**), GFAP (**K**), and C3 (**L**) in spinal cords from mice in (**A**). **M** Immunofluorescence images of Nestin (red) and SOX2 (green) on day 21 after injury in spinal cord lesion areas. All nuclei were stained with DAPI (blue). Scale bar, 50 μm. (**N**) Western blot bands displaying Nestin protein levels of spinal cord from mice 21st day after injury. **O** Quantification of NSCs in (**M**). **P** Semi-quantitative analysis of Nestin levels normalized to β-actin in (**N**). **Q** mRNA expression levels of Nestin in spinal cord 21 days after surgery. All data are presented as means ± SD (*n* = 5 mice per group). *p < 0.05; **p < 0.01; ***p < 0.001.

To corroborate the observed phenomena in vitro, we administered salidroside treatment along with corresponding pathway agonists in vivo. In consideration of the disappearance of microglia/macrophages infiltration in SCI mice approximately 2 weeks post injury [2, 4], we delayed the initiation of administration until the 16th day post injury in order to mitigate the impact of microglia/macrophages. Immunofluorescence analysis revealed that sustained treatment with a high dose of salidroside during the late stage of injury significantly attenuated the accumulation of glial scars and augmented the number of neurons in the injured area (Fig. 7B, D, E). Concurrently, the use of the STAT3 agonist colivelin antagonized the inhibitory effect of salidroside on glial scars, although its impact on neuronal recovery was comparatively modest. In contrast, the simultaneous use of the JNK agonist anisomycin did not significantly increase glial scars but substantially attenuated the neuroregenerative effect of salidroside. Consistent results were observed at the protein and mRNA expression levels in tissue samples (Fig. 7C, F–L). Moreover, the JNK agonist markedly impeded the increase of NSCs in the damaged area induced by salidroside, leading to reduced protein and mRNA expression levels of Nestin, while the STAT3 agonist exhibited no significant effect on NSC infiltration (Fig. 7M–Q). These findings suggest that the JNK pathway exerts a more pronounced influence on the proliferation and infiltration of NSCs. The JNK agonist inhibits neuronal repair by diminishing the number of NSCs while concurrently reducing glial scars. In contrast, the STAT3 agonist promotes the differentiation of NSCs into astrocytes, thereby exacerbating glial scars. It is essential to note the intricate cellular network present in the late stage of spinal cord injury (SCI), encompassing complex intercellular interactions among astrocytes, NSCs, and even T cells, NK cells, and B cells infiltrating spinal cord tissue [38–40]. Further validation necessitates in vivo gene editing of specific cell types.

### Salidroside inhibited the phosphorylation of JNK and STAT3 pathways by upregulating Sirt1

SIRT1, a histone deacetylase, plays a pivotal role in inhibiting the acetylation and phosphorylation of multiple signaling pathways [41–43]. Following SCI, the expression of Sirt1 typically decreases, and restoring its levels has been shown to be beneficial for SCI recovery [44]. Our investigation revealed that salidroside effectively restored Sirt1 expression levels. Importantly, the Sirt1 inhibitor EX527 not only counteracted the upregulation of Sirt1 by salidroside but also nullified the inhibitory effect of salidroside on STAT3 and JNK phosphorylation (Supplemental Fig. 6). This observation implies that salidroside may contribute to functional recovery post-SCI by enhancing Sirt1 expression and consequently inhibiting the phosphorylation of STAT3 and JNK pathways.

## Discussion

The current clinical approach to treating SCI in addition to surgery predominantly relies on glucocorticoid pulse therapy administered within the first 24 h post injury. However, this treatment exhibits inconsistent results and is often accompanied by significant adverse effects [2, 45, 46]. Therefore, the search for effective therapeutic interventions for SCI remains a pivotal area of research within the field of SCI management. Presently, research efforts in drug development for SCI primarily concentrate on early short-term anti-inflammatory treatments. These investigations encompass a wide array of interventions, ranging from various enzymes, natural compounds, small molecule drugs, to exosomes [47–50]. While these interventions have shown promising results in inhibiting secondary injury and reducing functional loss by controlling early inflammation, their efficacy is somewhat compromised by the diversity of patient injury mechanisms and logistical limitations associated with early administration [2, 51]. Given the protracted nature of SCI pathology lasting weeks or months and the subsequent slow process of regeneration and repair following early inflammation, exploring the long-term therapeutic impact of novel drugs, particularly in the later stages of SCI when neural regeneration becomes critical, is of practical significance. Here our research demonstrates that extended salidroside treatment substantially enhances functional recovery and tissue repair in SCI-afflicted mice.

**Fig. 8:**
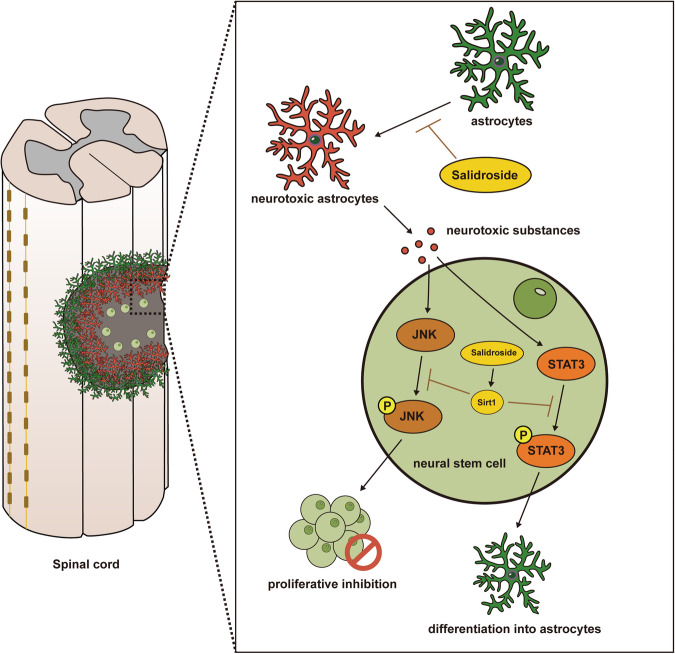
Salidroside promotes the repair of spinal cord injury by inhibiting astrocyte polarization, promoting neural stem cell proliferation and neuronal differentiation. Schematic model illustrating the possible mechanism of salidroside’s regulation of the late-stage spinal cord microenvironment. In the late stage of SCI, NSCs migrate into the lesion core. Astrocytes proliferate and form a glial scar that envelops the injured area. Some astrocytes polarize into neurotoxic A1 astrocytes, which inhibit NSCs proliferation by increasing JNK pathway phosphorylation and induce NSCs differentiation into astrocytes through STAT3 pathway activation by secreting neurotoxic substances (including TNF-a, IL-1β, IL-6, endothelin 1, excess lactate and γ-amino butyric acid, etc). Salidroside directly inhibits astrocyte proliferation and A1 polarization and acts directly on NSCs to mitigate proliferation inhibition and restore neuronal differentiation by reducing JNK and STAT3 phosphorylation levels possibly through upregulating Sirt1.

NSCs have been extensively studied for SCI treatment. These studies encompass intravenous or local NSC injections and implantation into the injury site with the aid of material-based scaffolds [52, 53]. Typically, NSC transplantation occurs during the later stages of SCI, aiming to evade the excessive inflammation and oxidative stress characteristic of the early stages [54, 55]. Additionally, this timeframe coincides with a critical period for the proliferation and differentiation of endogenous NSCs that migrate to the injury site [15, 16]. Nonetheless, the differentiation of NSCs into neurons remains relatively inefficient, signifying that strong impediments persist in the late-stage spinal cord microenvironment [32]. In the late stages of SCI, astrocytes become activated, proliferate, and transform into formidable glial scars, which, in turn, secrete extracellular matrix to bridge scar gaps, inhibiting neuronal reconnection [8, 9]. Recent investigations suggest that these astrocytes can further differentiate into neurotoxic variants, perpetually damaging nerve cells and hindering endogenous repair processes [12, 14]. Our research reveals that neurotoxic astrocytes hinder NSC proliferation and induce their differentiation into astrocytes during the late stage of SCI, thus exacerbating the glial scar. This underscores the presence of persistent injurious factors throughout the regenerative phase of SCI, demanding appropriate pharmacological interventions to enhance the spinal cord microenvironment. Our findings indicate that sustained administration of salidroside can suppress astrocyte proliferation and neurotoxic differentiation during the late stages of SCI. Furthermore, salidroside can directly affect NSCs. Through upregulating Sirt1, salidroside can inhibit astrocytic differentiation by repressing the STAT3 pathway and alleviate proliferation inhibition by dampening the JNK pathway (Fig. 8). In comparison to many synthetic small molecule drugs, salidroside, as a natural compound, presents lower tissue and cell toxicity, with the ability to comprehensively influence multiple signaling pathways for a more holistic therapeutic effect. However, it’s crucial to emphasize that the efficacy of natural compounds, including salidroside, necessitates appropriate long-term treatment. We propose that a judicious and sustained regimen of salidroside after SCI may bolster functional recovery by promoting endogenous regeneration and mitigating factors inhibitory to nerve regeneration. Additionally, salidroside may hold potential as an adjunctive therapy for stem cell transplantation.

Salidroside, beyond its influence on microglia/macrophage polarization, also attenuates secondary injury by suppressing the secretion of inflammatory factors by astrocytes [24, 26]. Inflammatory pathways such as NF-κB and Notch not only regulate the secretion of inflammatory substances but also impact cell differentiation and proliferation through pathways like STAT3 and JNK in inflammatory cells [14, 56, 57]. While NSCs do not generate inflammation, similar regulatory cascades governing their differentiation and proliferation may exist. Our investigation focused on salidroside’s effects on STAT3 and JNK phosphorylation, which represent terminal positions within their respective pathways. However, it is essential to acknowledge the possibility of extensive cellular pathway alterations upstream. Thus, comprehensive upstream pathway analysis is still needed to further elucidate the precise mechanism of salidroside action on NSCs.

Astrocytes significantly influence the regeneration and repair processes post-SCI^9^. These cells, once activated, not only contribute to the formation of glial scars but also release inflammatory factors capable of damaging nerve cells [11, 12]. The detrimental impact of astrocytes on nerve cells is more intricate than initially perceived, and numerous gaps persist in understanding the specific underlying mechanisms [9]. Recent studies have shed light on TAFA4 (also known as FAM19A4), a chemokine-like ligand primarily expressed in sensory neurons within the spinal dorsal horn [25]. TAFA4 serves as a marker for C-low-threshold mechanoreceptors and is closely associated with neuroinflammation and neuronal electrophysiological activity [58, 59]. While upregulation of TAFA4 expression has been observed in activated macrophages and monocytes, its expression in astrocytes remains unexplored [60]. Given the pivotal role of astrocytes in neuroinflammation and electrophysiological function as indicated by recent research [61, 62], investigating whether salidroside modulates SCI regeneration by regulating TAFA4 expression in astrocytes represents a promising avenue for further inquiry. Furthermore, astrocytes regulate nerve cell fate through various metabolic processes, including amino acid, fatty acid, and glucose metabolism [62–64]. Exploring the potential of salidroside to modulate these metabolic pathways is essential for a comprehensive understanding of its effects on nerve cell regeneration and repair post-SCI.

Nevertheless, our study has inherent limitations. Complex signal networks and intercellular interactions within the organization make it challenging to accurately extrapolate in vitro experimental results to in vivo experiments. For instance, glial scars originate not only from astrocytes differentiated from NSCs but also from the proliferation and activation of resident astrocytes in tissues. Additionally, after peripheral administration, salidroside may exert its regulatory effect on SCI through peripheral pathways, potentially affecting innate and adaptive immunity. Furthermore, the efficiency of salidroside crossing the blood-brain barrier is a concern. the repair of the blood-brain barrier may reduce salidroside efficacy, while overdosage can increase the risk of organ toxicity. Therefore, a crucial avenue of future research involves the development of carriers designed to target nerve tissues and facilitate the passage of salidroside across the blood-brain barrier, as an in vivo transport method for peripheral salidroside usage. Gene editing in animals is imperative for more accurate observations of salidroside effects on specific cell types and signaling pathways. Additionally, the prospect of combination therapy involving salidroside and NSC transplantation warrants exploration to ascertain its effects and delineate treatment protocols.

### Supplementary information

Supplementary Information

Original full length western blots

## Data Availability

The data that support the findings of this study are available from the corresponding author upon reasonable request.
