# Supplementary material for: Salidroside promotes the repair of spinal cord injury by inhibiting astrocyte polarization, promoting neural stem cell proliferation and neuronal differentiation
Source: Cell Death Discov. 2024 May 9;10:224. doi: 10.1038/s41420-024-01989-2 (PMC11082153; doi:10.1038/s41420-024-01989-2)
Supplement: Supplementary file 1 — Supplementary Information [file 41420_2024_1989_MOESM1_ESM.doc]

**Summary of supplementary information**

1. Supplemental Fig 1: A schematic representation of *in vitro* cell experiments.
2. Supplemental Fig 2: Identification of primary NSCs.
3. Supplemental Fig 3: Drug toxicity experiments including H&E staining and Serum biochemical analysis.
4. Supplemental Fig 4: Representative immunofluorescence images and mRNA levels of macrophages in spinal cord 7 days after SCI.
5. Supplemental Fig 5: Representative immunofluorescence images of MBP in spinal cord 28 days after SCI.
6. Supplemental Fig 6: Western blot bands displaying total protein levels and phosphorylated protein levels of primary NSCs under ACM stimulation for 24 hours in the presence or absence of Sal (100 μM) or EX527 (10 μM).


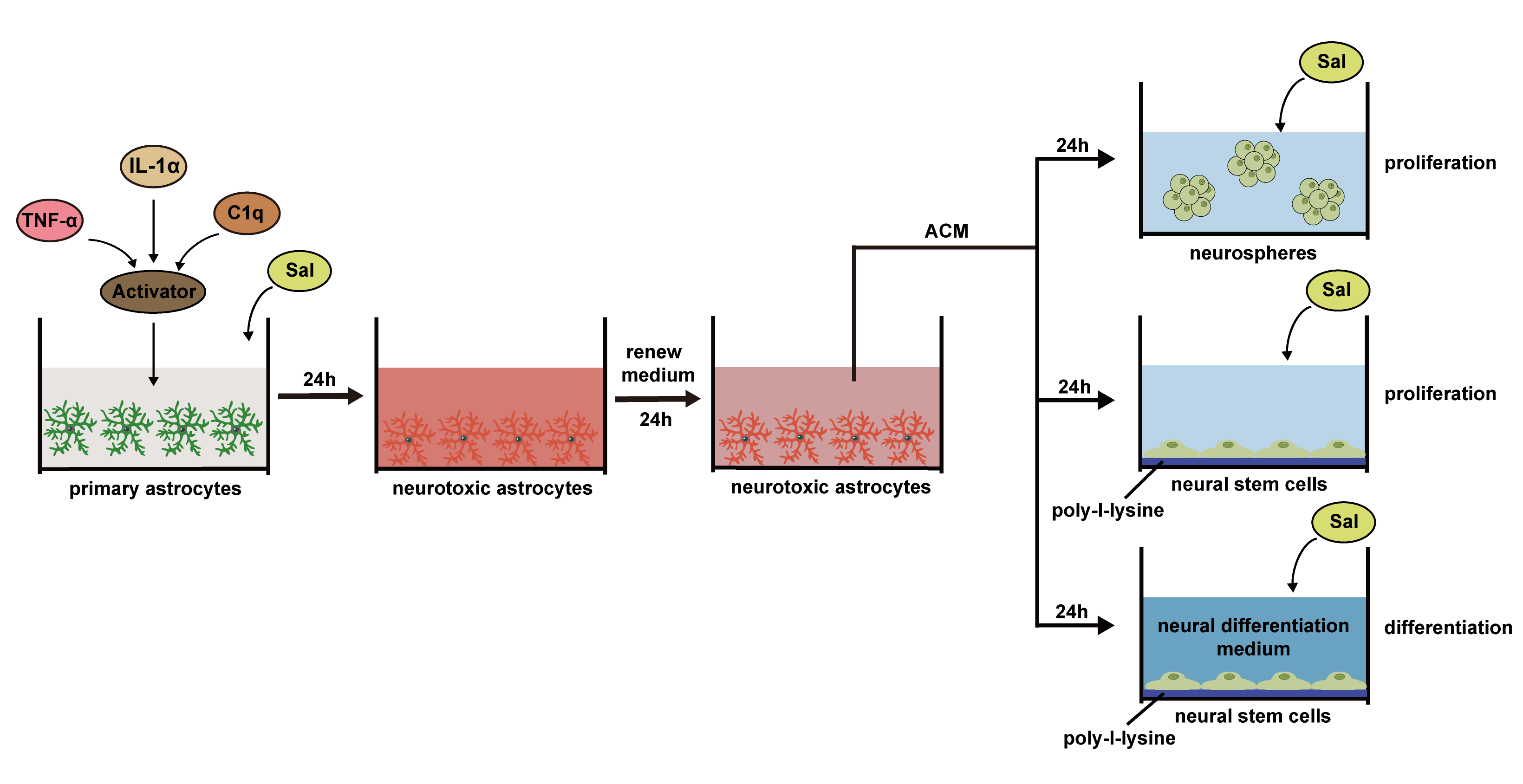


Supplemental Fig. 1. A schematic representation of *in vitro* cell experiments. IL-1α (3 ng/mL), TNFα (30 ng/mL), and C1q (400 ng/mL) (the mixture was called Activator) were added simultaneously to the medium and cultured for 24h to induce neurotoxic A1 astrocytes. Salidroside (Sal) could be added at the same time to block the effect of the Activator. The culture medium of neurotoxic astrocytes was then replaced with a new complete medium to remove the effect of the Activator and culture was continued for 24h. The supernatant was collected (astrocyte-conditioned medium, ACM) for stimulation of NSCs, including suspended neurospheres and adherent single cells. Salidroside or inhibitors could be added simultaneously to suppress the effect of the ACM.


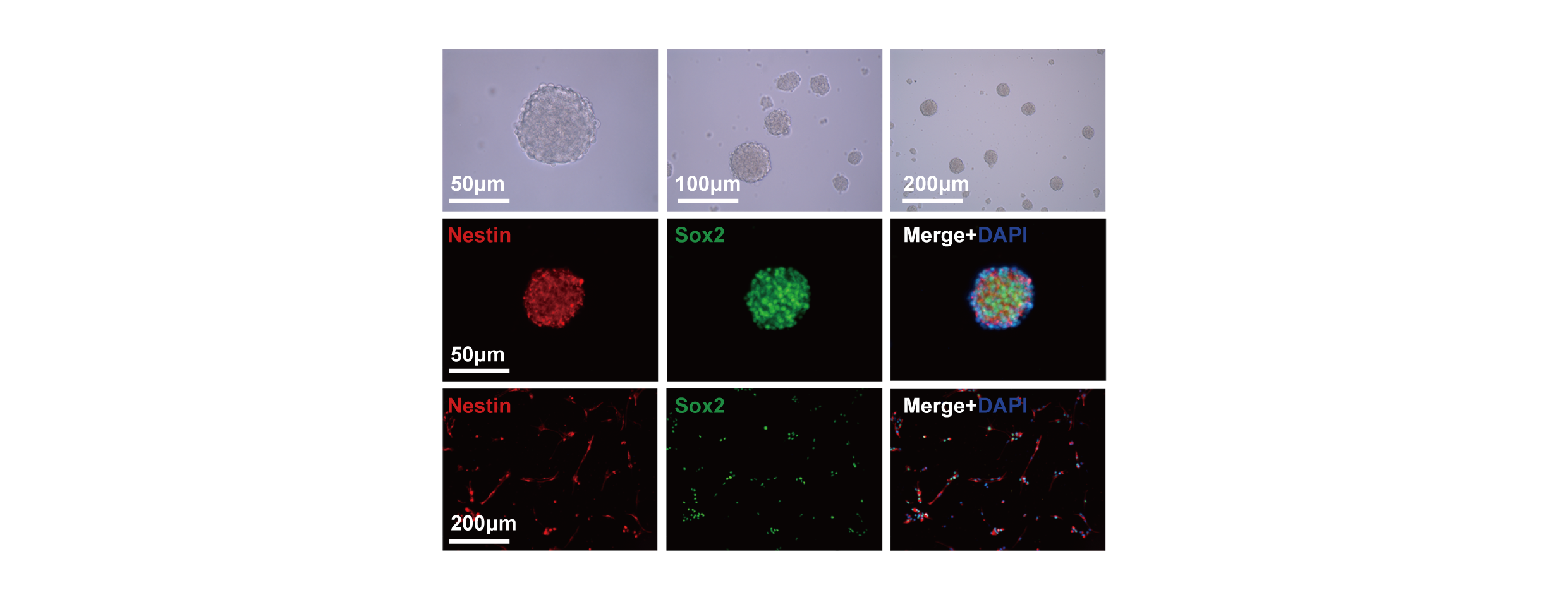


Supplemental Fig. 2. Identification of primary NSCs. Row 1: Bright-field photographs of neurospheres at different magnifications. Row 2: Representative images of Nestin (red) and Sox2 (green) immunofluorescence staining of neurospheres. Row 3: Representative images of Nestin (red) and Sox2 (green) immunofluorescence staining of adherent single NSCs. All cell nuclei were stained with DAPI (blue).


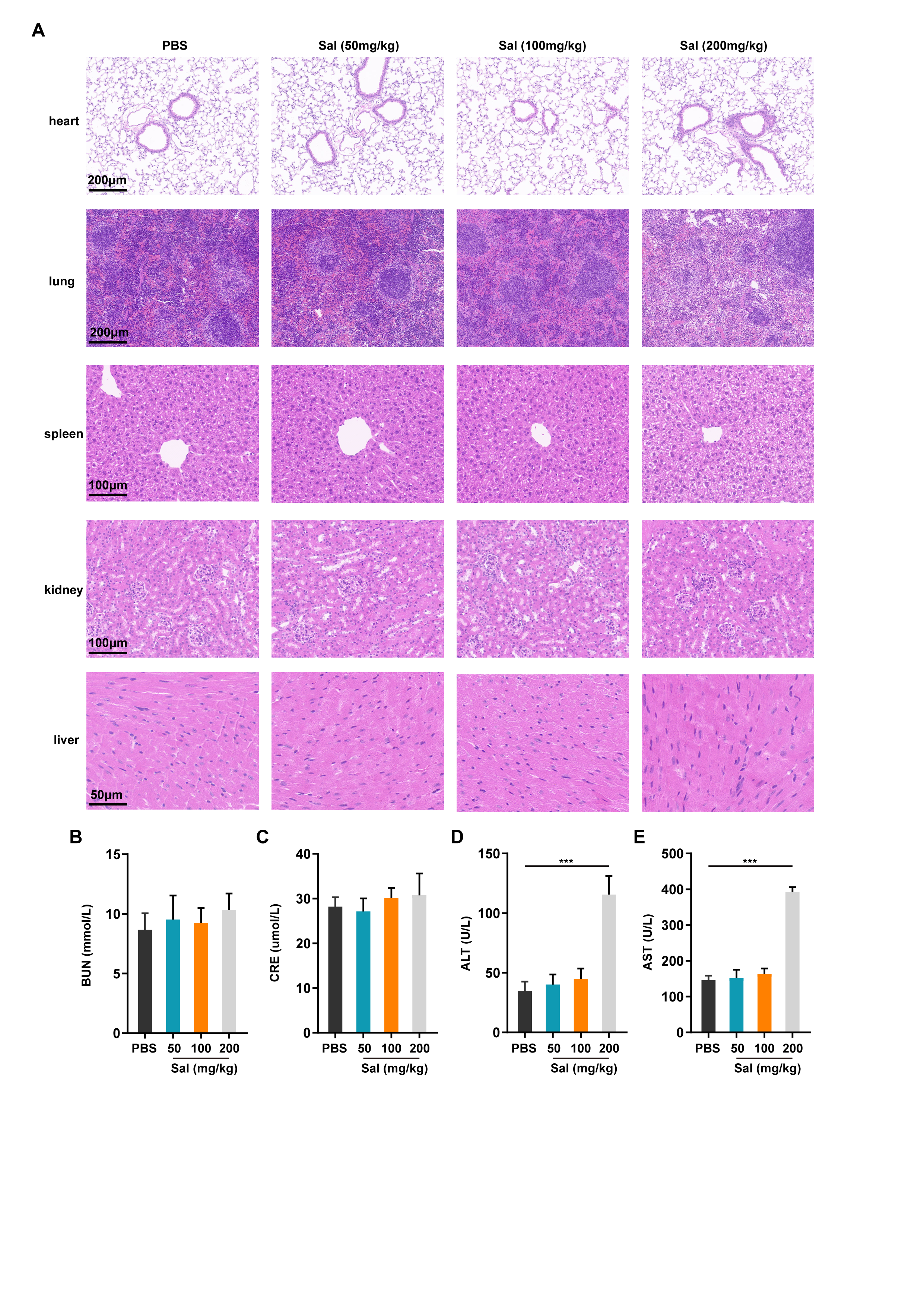


Supplemental Fig. 3. (A) Representative images of H&E staining showing histopathology of the liver, lung, kidney, heart and spleen. (B-E) Serum biochemical analysis of systemic toxicity. (B) BUN, blood urea nitrogen; (C) CRE, creatinine; (D) ALT, alanine transaminase; (E) AST, aspartate transaminase. All data are presented as means ± SD (n = 4 mice per group). **p*< 0.05; ***p* < 0.01; ****p* < 0.001.


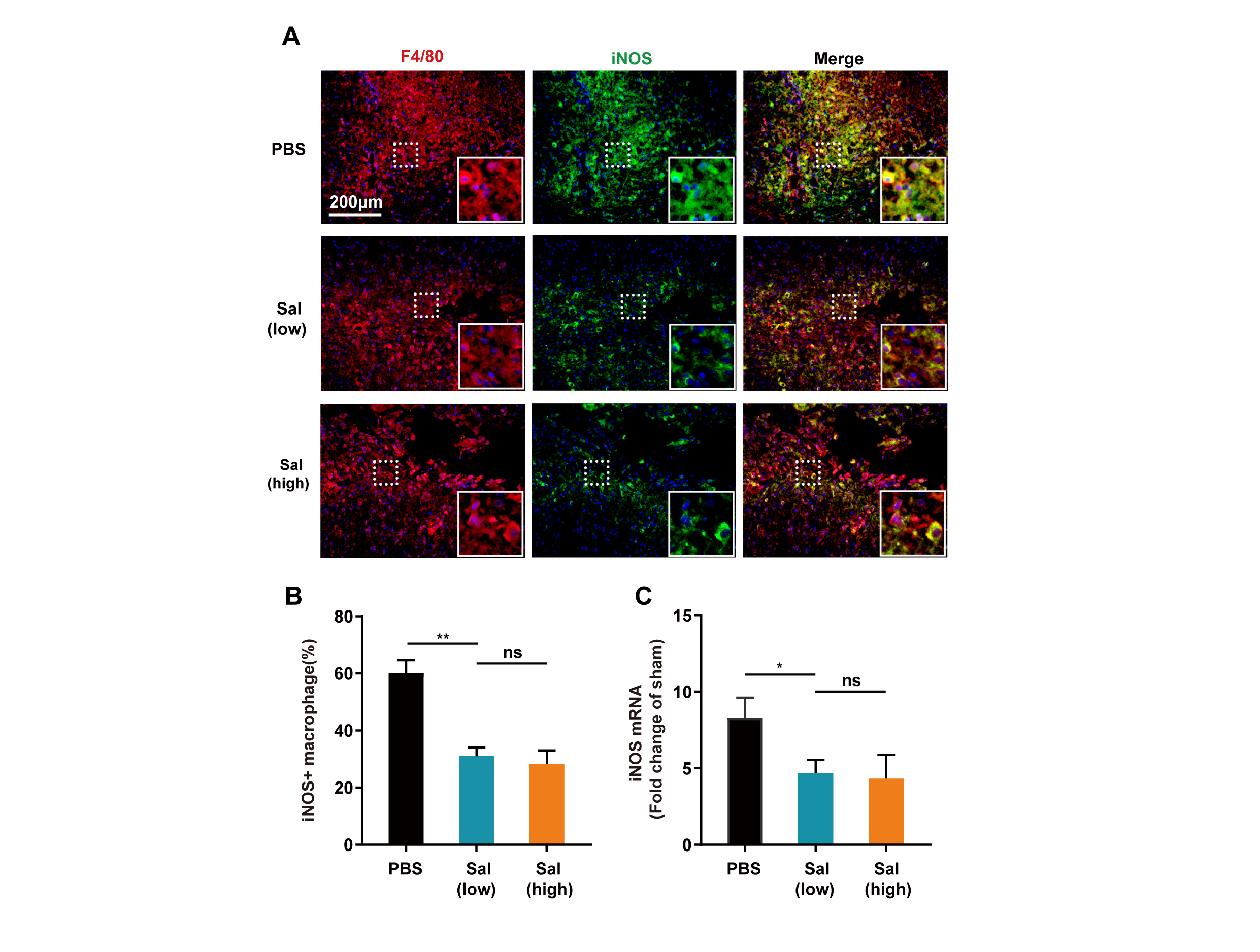


Supplemental Fig. 4. (A) Representative immunofluorescence images of spinal cord 7 days after SCI. Macrophages were stained with iNOS (green) and labeled by F4/80 (red). Nucleus was counterstained with DAPI. Scar bar, 200 μm. (B) Quantitative analysis of the ratio of iNOS+ Macrophages around the lesion areas in (A). (C) mRNA expression levels of iNOS in spinal cord 7 days after surgery. All data are presented as means ± SD (n = 5 mice per group). **p*< 0.05; ***p* < 0.01; ****p* < 0.001.


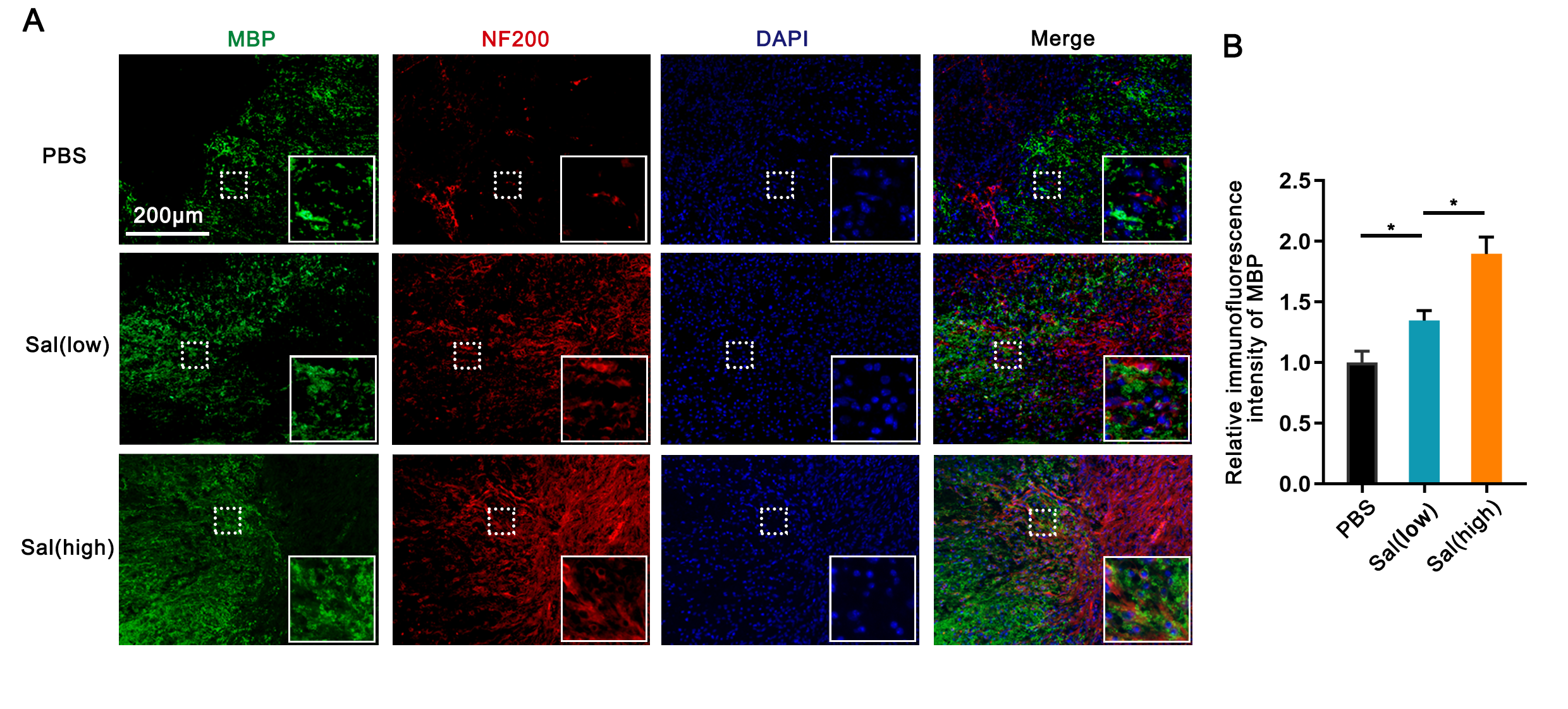


Supplemental Fig. 5. (A) Immunofluorescence images of NF200 (red) and MBP (green) staining on the 28^th^ day after SCI. All nuclei were stained with DAPI (blue). Scale bar, 200 μm. (E) Semi-quantification of MBP intensity in (A). All data are presented as means ± SD (n = 5 mice per group). **p*< 0.05; ***p* < 0.01; ****p* < 0.001.


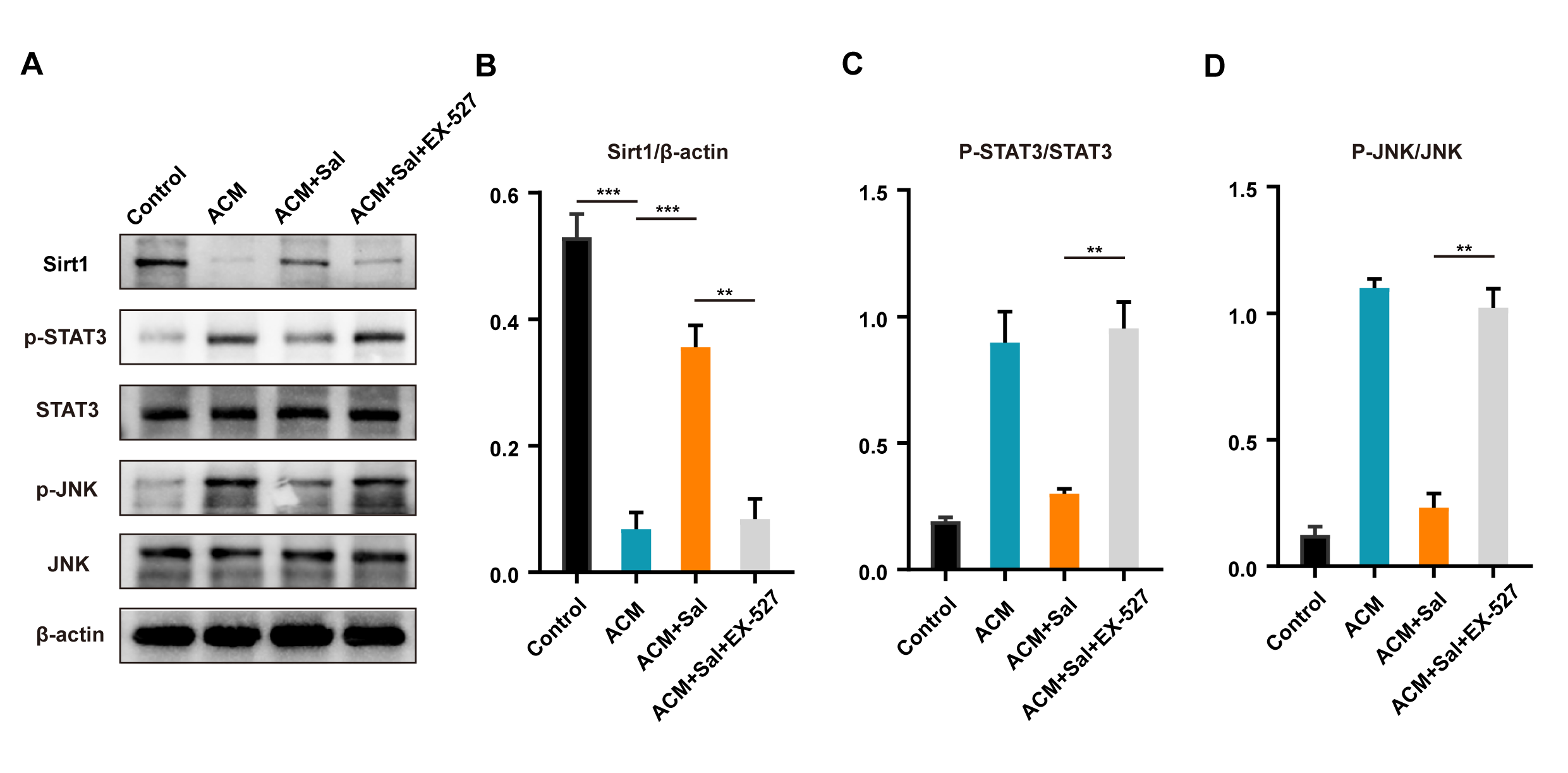


Supplemental Fig. 6. (A) Western blot bands displaying total protein levels (STAT3, JNK, and Sirt1) and phosphorylated protein levels (p-STAT3, p-JNK) of primary NSCs under ACM stimulation for 24 hours in the presence or absence of Sal (100 μM) or EX527 (10 μM). (B) Semi-quantitative analysis of protein levels in (A). All data are presented as means ± SD (n = 6 / group). **p*< 0.05; ***p* < 0.01; ****p* < 0.001.
