# Supplementary figures and images for: Salidroside promotes the repair of spinal cord injury by inhibiting astrocyte polarization, promoting neural stem cell proliferation and neuronal differentiation
Source: Cell Death Discov. 2024 May 9;10:224. doi: 10.1038/s41420-024-01989-2 (PMC11082153; doi:10.1038/s41420-024-01989-2)

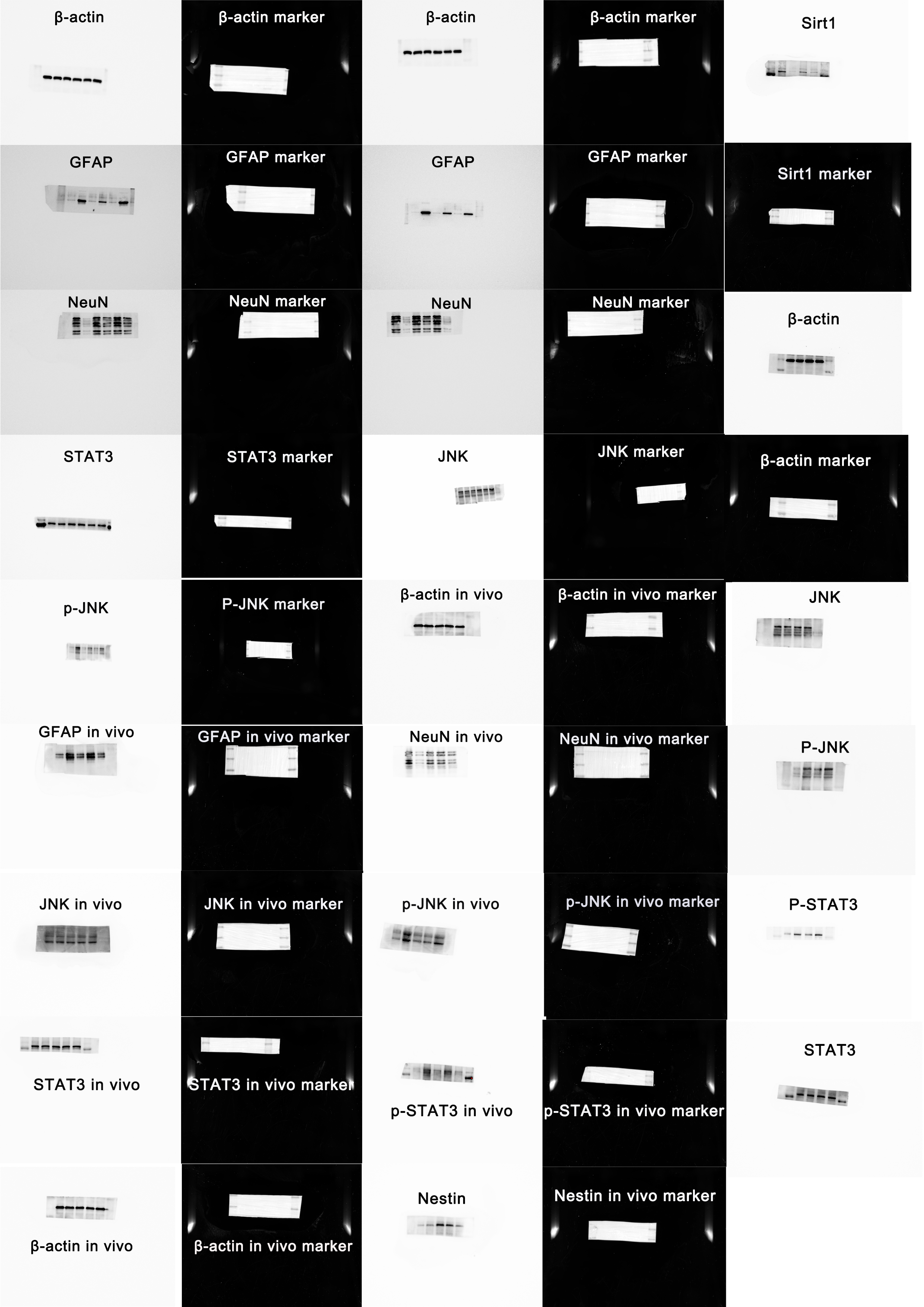

Supplement: Supplementary file 2 — Original full length western blots [file 41420_2024_1989_MOESM2_ESM.tif]
